# The Curative and Prophylactic Effects of Xylopic Acid on *Plasmodium berghei* Infection in Mice

**DOI:** 10.1155/2013/356107

**Published:** 2013-07-18

**Authors:** J. N. Boampong, E. O. Ameyaw, B. Aboagye, K. Asare, S. Kyei, J. H. Donfack, E. Woode

**Affiliations:** ^1^Department of Biomedical and Forensic Sciences, University of Cape Coast, Cape Coast, Ghana; ^2^Department of Optometry, School of Physical Sciences, University of Cape Coast, Cape Coast, Ghana; ^3^Department of Biomedical Sciences, Faculty of Sciences, University of Dschang, P.O. Box 067, Dschang, Cameroon; ^4^Department of Pharmacology, Faculty of Pharmacy and Pharmaceutical Sciences, College of Health Sciences, KNUST, Kumasi, Ghana

## Abstract

Efforts have been intensified to search for more effective antimalarial agents because of the observed failure of some artemisinin-based combination therapy (ACT) treatments of malaria in Ghana. Xylopic acid, a pure compound isolated from the fruits of the *Xylopia aethiopica,* was investigated to establish its attributable prophylactic, curative antimalarial, and antipyretic properties. The antimalarial properties were determined by employing xylopic acid (10–100 mg/kg) in ICR mice infected with *Plasmodium berghei*. Xylopic acid exerted significant (*P* < 0.05) effects on *P. berghei* infection similar to artemether/lumefantrine, the standard drug. Furthermore, it significantly (*P* < 0.05) reduced the lipopolysaccharide- (LPS-) induced fever in Sprague-Dawley rats similar to prednisolone. Xylopic acid therefore possesses prophylactic and curative antimalarial as well as antipyretic properties which makes it an ideal antimalarial agent.

## 1. Introduction

**Figure 1:**
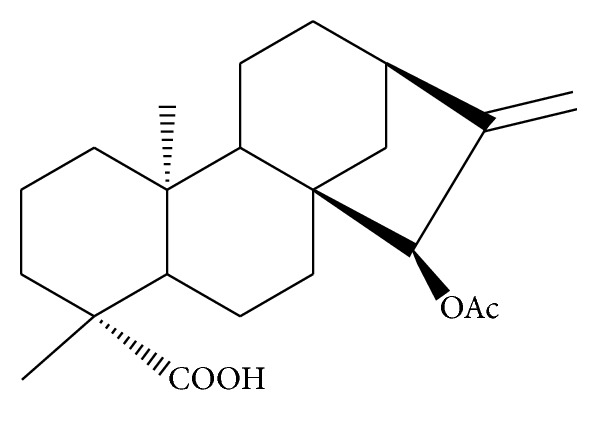
Chemical structure of xylopic acid.

Malaria, caused by *Plasmodium* parasite, is a leading poverty-associated disease that undermines the development of countries. The numbers of disease cases and deaths was 225 million and 781 000 respectively in 2009 [1]. Children under five years and pregnant women (vulnerable groups) succumb to the devastating effects of the disease making the disease a major global infectious disease. Chemotherapy has ultimately been the central tool for management of malaria, and combination of drug regimens has become the practice of choice because of their increased therapeutic efficacy over monotherapy and other benefits which include decreased cytotoxicity and delay or prevention of the development of drug resistance [2]. *Plasmodium falciparum *(Pf), the most lethal malaria pathogen, has developed resistance to some antimalarials [3]. This makes it imperative to search for newer, more effective antimalarial agents. Plants have served as reliable sources of drugs especially antimalarials [4]. The fruits of *Xylopia aethiopica* are used traditionally for the treatment of malaria but the active principle(s) responsible for the observed antimalarial effect of the extract is still not known [5, 6]. Xylopic acid, a kaurene diterpene, occurs as the major constituent in the fruits of *Xylopia aethiopica* and is reported to possess analgesic properties [7]. Xylopic acid, unlike kaurenoic acid, has no cytotoxic effect against human cancer cells [8], making the compound a safe one for the treatment of diseases where selective toxicity towards the parasite is highly needed. In the light of the above, xylopic acid was evaluated for its antimalarial and antipyretic properties. The structure of the xylopic acid is shown below (Figure 1).

## 2. Materials and Methods

### 2.1. Extraction and Purification of Xylopic Acid (15*β*-Acetoxy-(−)-kaur-16-en-19-oic Acid)

The extraction process was carried out as described elsewhere [7]. Briefly, 0.36 kg of the fruit of *Xylopia aethiopica *was pulverized and placed in cylindrical jars. This was soaked with 5 L of petroleum ether (40–60°C) and allowed to stand for three days. The petroleum ether was drained and concentrated using rotary evaporator at a temperature of 50°C. Ethyl acetate was added to the concentrate to facilitate the crystallization of xylopic acid. Crystals (xylopic acid) formed after the concentrate had been allowed to stand for three days and were washed with petroleum ether at 40–60°C repeatedly until all unwanted materials had been removed. Crude xylopic was purified in 96% ethanol. The yield of the xylopic acid was 1.41%. The purity of the isolated xylopic acid was 95%.

### 2.2. Chemicals and Test Agents

The lipopolysaccharide (LPS), ethanol, petroleum ether, and ethyl acetate used for the extraction were purchased from Sigma-Aldrich Inc., St. Louis, MO, USA. Artemether/Lumefantrine (A-L) was obtained from Ajanta Pharma Ltd., Maharashtra, India, sulfadoxine/pyrimethamine was obtained from Maxheal Laboratories Pvt. Ltd. Gujarat, India, and prednisolone from (Anhui Medical Co. Ltd). 

### 2.3. Animals

Male ICR mice (25–30 g) and Sprague-Dawley (150–200 g) rats of both sexes were housed in the animal facility of the Department of Biomedical and Forensic Sciences, University of Cape Coast (UCC). The animals were housed in groups of five in stainless steel cages (34 × 47 × 18 cm) with soft wood shavings as bedding, fed with normal commercial pellet diet (AGRICCARE, Kumasi), given water *ad libitum*, and maintained under laboratory conditions. All procedures and techniques used in these studies were in accordance with the National Institute of Health Guidelines for the Care and Use of Laboratory Animals [9]. All protocols used were approved by the Departmental Ethics Committee.

#### 2.3.1. Source of Rodent Parasite (*Plasmodium berghei* NK65)

The rodent parasite was obtained from Noguchi Memorial Institute for Medical Research, University of Ghana, Legon, Ghana, and maintained alive in mice by continuous intraperitoneal passage in mice after every 6 days [10]. The reinfected mice were kept in the animal house of the Department of Biomedical and Forensic Sciences. 

#### 2.3.2. Inoculation of Parasite

Total inoculum concentration of 60 × 10^6^ of *P. berghei* parasitized erythrocytes per mL was prepared. This was carried out by determining the parasite density of the *Plasmodium berghei-*infected mice. The blood obtained from the infected mice was diluted appropriately with EDTA-phosphate buffer saline (PBS) and subsequently washed with PBS. Each mouse was intraperitoneally inoculated on day 0 with 0.2 mL of infected erythrocytes containing 1 × 10^6^ *P. berghei *parasitized red blood cells. 

### 2.4. Effect of Xylopic Acid on Established *Plasmodium berghei* Infection

To evaluate the curative antimalarial properties of xylopic acid on established *Plasmodium berghei *infection, thirty male mice were each inoculated with 1 × 10^6^ *P. berghei *on the first day [11]. The mice were assigned to five groups (*n* = 6). Seventy-two hours later, the animals were treated once daily with three doses of xylopic acid (10, 30, and 100 mg/kg *p.o.*) (groups 1–3), 4 mg/kg *p.o.* of artemether/lumefantrine (A-L) (standard drug: group 4), and 10 mL/kg *p.o.* normal saline (group 5) for 5 days. To determine the daily parasitaemia level, about three drops of blood were collected from the tail of each mouse and smeared onto a microscope slide to make a thin film. The thick film was prepared from two drops of blood obtained from the tail of the mice. The smears were fixed in absolute ethanol and stained with 10% Geimsa stain, and examined microscopically (×100 magnification). The parasitaemia was determined by counting infected erythrocytes in hundred fields, divided by the total erythrocytes in the hundred fields then multiplied by hundred. On the twelfth day (D 13), two animals from each treatment group were sacrificed, and the liver were taken for histopathological assay. The tissue was embedded in paraffin; 8 *μ*m sections were cut on a microtome (Bright 5040, Bright instrument company Ltd., England) and processed for routine haematoxylin-eosin staining. Slides of tissue sections were observed using trinocular clinical light microscope with a digital camera (Olympus CX1, Japan) connected to a computer. Micrographs of the tissue were generated using the ×10 objective lens for further analysis. The mean survival time of the mice in each treatment group was determined over a period of 30 days.

### 2.5. Prophylactic Activity of Xylopic Acid on *P. berghei* Infection

Xylopic acid was further assayed for its prophylactic activity against *P. berghei* infection using the method described by Peters [12]. The mice were randomly assigned to five groups (*n* = 6) and pretreated orally with 10, 30, and 100 mg/kg/day of xylopic acid, 1.2 mg/kg/day sulfadoxine/pyrimethamine (SP, the reference drug), and 10 mL/kg/day of normal saline. The treatment was continued for 3 consecutive days. On the fourth day, all mice were infected with 1 × 10^6^ *P. berghei*, and seventy-two hours later, blood smears were prepared from the tail. The parasite density and % chemosuppression for all the treatment groups were determined.

### 2.6. Lipopolysaccharide-Induced Fever

The method of Santos and Rao [13] was used with slight modification for the assessment of the antipyretic activity of xylopic acid. Rats were fasted overnight prior to induction of fever, and water was given *ad libitum*. Rectal temperature was measured using a lubricated ECT-1 digital thermometer (Estar Electronic And Instrument Co., Ltd., Zhejiang, China) inserted 3 mm deep into the rectum of the rats. Fever was induced by injecting intramuscularly 1 mg/kg of LPS into the right thigh of each rat. Rectal temperature was measured again, and animals that showed an increase in temperature of 0.5°C and more were selected for the study. The animals with fever were put into five groups (*n* = 6) and were treated with three doses of xylopic acid (10–100 mg/kg), 30 mg/kg of prednisolone, or 1 mL/kg of normal saline solution (the control), orally, two hours after LPS-induced fever. Rectal temperature was measured after 1 h of the treatments. 

### 2.7. Statistical Analysis

GraphPad Prism for Windows version 4.03 (GraphPad Software, San Diego, CA, USA) was used for all statistical analyses, and *P* < 0.05 was considered statistically significant. All data were expressed as mean ± SEM (duplicate measurement). The time-course curves were subjected to two-way (treatment × time) repeated measures analysis of variance (ANOVA) with Bonferroni's *post hoc* test. The column graphs were subjected to one-way analysis of variance (ANOVA) with Tukey's *post hoc* test.

## 3. Results

### 3.1. Curative Activities Of Xylopic Acid and A-L on *P. berghei* Infection in Mice

**Figure 2:**
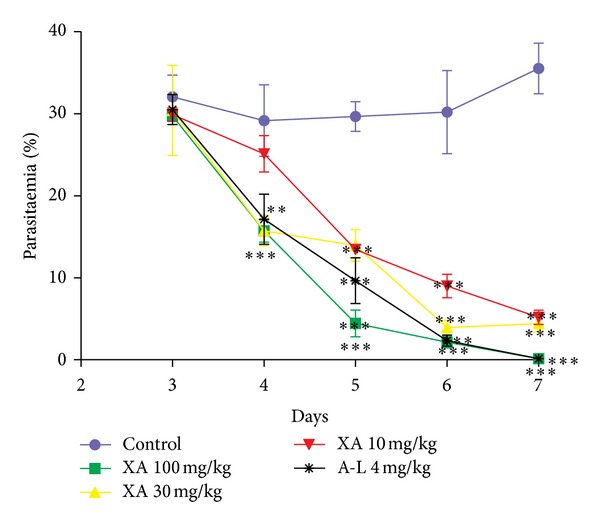
Curative effect of xylopic acid and A-L on the time-course curve of *Plasmodium berghei *infection in mice. Data is presented as mean ± SEM. ***P < 0.001, **P < 0.01 compared to vehicle-treated group (two-way ANOVA followed by Bonferroni's* post hoc *test).

Xylopic acid and A-L reduced the parasitaemia significantly from the first day of treatment to the final day. Again, all of the treatments resulted in relatively increased the survival time of the mice compared to the control (Table 1). Xylopic acid significantly (*P* < 0.0001) reduced the level of parasitaemia from day one after treatment and achieved the highest effect on the last day (Figure 2). The % parasitaemia decreases by the 10 mg/kg xylopic acid were 6.7%, 13.9%, 54.7%, 70%, and 85.4 from day 1 to day 5 after treatment, respectively. The 30 mg/kg xylopic acid also produced % parasitaemia reductions of 5.2%, 46.1%, 53%, 86.9%, and 87.6% from day 1 to day 5 after treatment, respectively. Similarly, the % parasitaemia decreases by the 100 mg/kg xylopic acid were 7.4%, 46.1%, 84.8%, 92.8%, and 99.6% from day 1 to day 5 after treatment, respectively, (Figure 2). 

 The standard antimalarial drug A-L (4 mg/kg) produced a % of parasitaemia reductions of 1.1%, 35.2%, 63.6%, 91.7%, and 99.6% from day 1 to day 5 after treatment, respectively, (Figure 2).

The highest dose of xylopic acid (100 mg/kg) and 4 mg/kg A-L produced a maximum % 99.6% chemosuppression on the last day. The % chemosuppression of A-L was, however, 1.1 times greater than the maximum % chemosuppression produced by 30 mg/kg dose of xylopic acid and 1.2 times greater than the maximum % chemosuppression produced by the lowest dose of xylopic acid. The % chemosuppression of A-L was not statistically significant compared to the % chemosuppression produced by the various doses of xylopic acid (Table 1).

**Figure 3:**
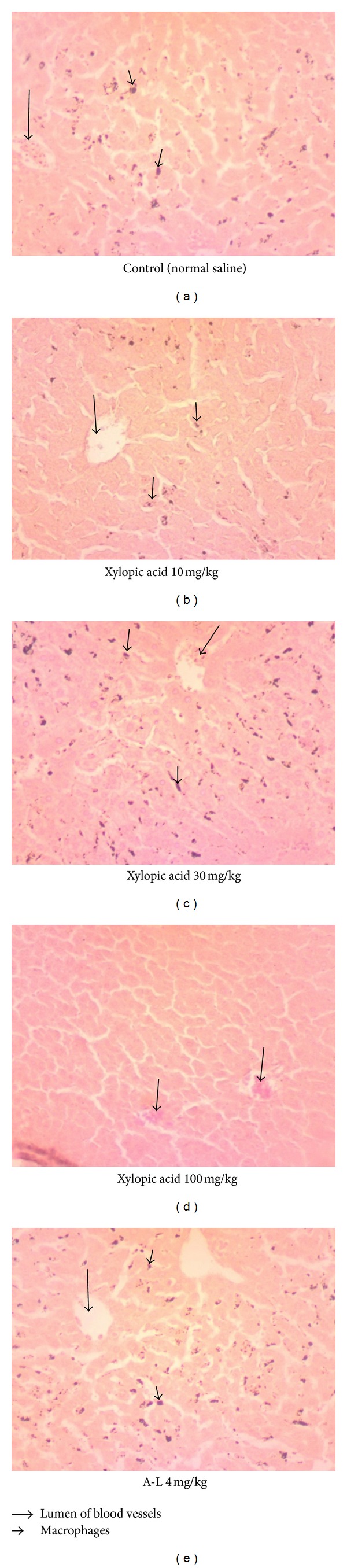
Photomicrographs of liver cells of animals treated with xylopic acid (10–100 mg/kg), artemether/lumefantrine (A-L) 4 mg/kg, and normal saline showing some Kupffer cells in the hepatocytes and *P. berghei*-infected red cells in the lumen of blood vessels.

Histopathological assessments of the hepatocytes reveal high levels of Kupffer cells in all the treated groups of animals except the mice treated with 100 mg/kg of xylopic acid. Parasites were observed in the lumen of the blood vessels of liver sections of the normal saline and middle dose of xylopic acid-treated groups but were barely seen in the lumen of A-L and the lowest and highest doses of xylopic acid-treated mice (Figure 3). 

### 3.2. Prophylactic Activities of Xylopic Acid and SP on *P. berghei* Infection in Mice

**Figure 4:**
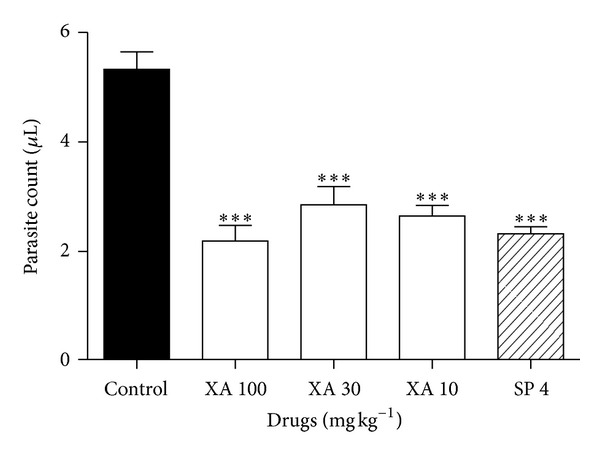
Prophylactic effects of xylopic acid and SP on *Plasmodium berghei *infection in mice. ***P < 0.001, compared to vehicle-treated group (One-way ANOVA followed by Tukey's* post hoc *test).

Xylopic acid exhibited significant (*P* < 0.05) prophylactic activity against *Plasmodium berghei  in vivo* at all of the three doses tested (Figure 4), seen as reduction in parasite count compared to the vehicle-treated group. The % chemosuppressive effect seen at the highest dose employed was 59.4%. 

### 3.3. Lipopolysaccharide-Induced Pyrexia

**Figure 5:**
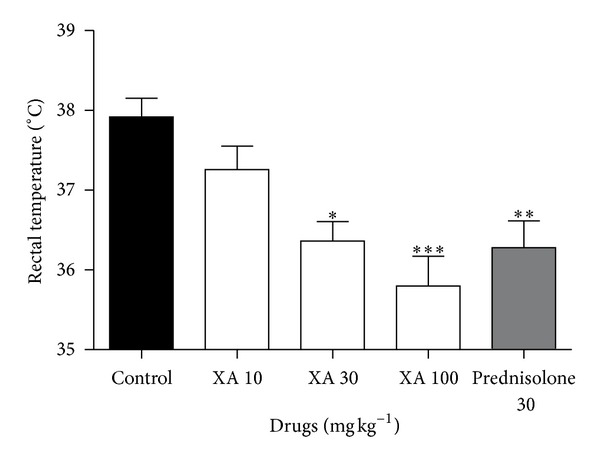
Antipyretic effects of xylopic acid (10–100 mg/kg) and prednisolone (10 mg/kg) on LPS-induced pyrexia in rats. Data plotted are means ± SEM. *P ≤ 0.05, **P ≤ 0.01, ***P ≤ 0.001; the level of significance of rectal temperature reduction compared to the normal saline-treated group (One-way ANOVA followed by Tukey's *post hoc* test).

Xylopic acid (30 and 100 mg kg^−1^) reduced significantly (*P* < 0.05) lipopolysaccharide-induced fever in rats (Figure 5). Prednisolone used as positive control also significantly reduced (*P* < 0.05) lipopolysaccharide-induced fever in rats (Figure 5). 

## 4. Discussion

Malaria infections are complicated syndromes involving many inflammatory responses which may enhance cell-to-cell interaction (cytoadherence), cell stimulation involving malaria-derived antigens/toxins and host-derived factors such as cytokines. Moderate amounts of cytokines are though good for the host causes fever [14]. Clinically, it is crucial to reverse the effects of both toxins and cytokines to prevent further complications of malaria. This makes xylopic acid an ideal agent for malaria treatment because it exerted curative and prophylactic properties on *P. berghei*-induced malaria in mice as well as antipyretic activities in rats. The inflammatory condition of malaria is charcterised by free radical generation, activation of phospholipase activity resulting in generation of eicosanoids such as prostaglandins and other cytokines (TNF, IFN-*γ*, and IL-1*β*). These inflammatory mediators as well as parasite sequestration are responsible for the disease. It has been suggested that the cytokines upregulate the expression of adhesion molecules such as ICAM-1 that is involved in the binding of the parasitized red blood cells to the vascular endothelium [15]. The curative antiplasmodial properties of xylopic acid may be due to the inhibition of the production and/or release of these inflammatory mediators associated with malaria. Indeed, xylopic acid has analgesic properties [7] and preliminary data in our laboratory also indicate that xylopic acid possesses potent anti-inflammatory properties. In addition, the curative effect may be attributed to its direct cytotoxic effect on the parasites in a mechanism similar to the A-L combination. A-L is an oral fixed dose combination of artemether (20 mg) and lumefantrine (120 mg). Artemether exerts its antimalaria properties by interference with parasite transport proteins, disruption of parasite mitochondrial function, inhibition of angiogenesis, and modulation of host immune function [16]. Lumefantrine, an aryl-amino alcohol, prevents detoxification of heam, resulting in parasite death from the toxic heam and free radicals [17, 18]. It is worth noting that xylopic acid completely eradicated parasites from the blood of the mice similar to the standard A-L. Xylopic acid again at the highest dose to a greater extent destroyed the parasites circulating in the lumen of the blood vessels of the liver [19]. The macrophages present in the liver sections of the control and low doses of xylopic acid-treated mice could be due to inflammatory processes induced by the circulating parasite [19]. The absence of macrophages in the liver sections of the highest dose of xylopic acid could be attributed to the complete elimination of the parasites from circulation [19]. Xylopic acid and A-L both prolonged the survival times of the mice and this could be attributed to the high parasitaemia clearance (reduced parasite burden) observed for these drugs [18]. 

Xylopic acid showed comparable efficacy to SP in the prophylaxis assay. This indicates the nonselectivity of xylopic acid on the stages of malaria parasite. It is not clear how xylopic acid exerts prophylactic activity on *P. berghei* infection but it may be inhibiting the multiplication of the parasites as well as direct cytotoxic effect on the parasites [16]. It may modulate the membrane properties of the erythrocytes preventing parasite invasion [15]. SP used in this study exerts prophylactic activities via the inhibition of dihydropteroate synthetase and dihydrofolate reductase enzymes of the parasites [20]. Generally, prophylactic antimalarial drugs work by disrupting the initial development of malaria parasites in the liver (causal activity). They may act by suppressing the emergent asexual blood stages of the parasite (suppressive activity) or by preventing the relapses induced by the latent liver forms (hypnozoites) [21]. Xylopic acid can therefore be used for malaria prophylaxis as well as a curative agent such as atovaquone/proguanil (Malarone), a drug approved in USA for malaria treatment and prophylaxis [22]. Although the rodent model presents with some limitations, it has successfully been validated through the identification of several conventional antimalarials including the currently used antimalarials, halofantrine, and the artemisinin derivatives [23].

Proinflammatory mediators, IL-2, and PGE_2_, are among the important mediators of LPS-induced pyrexia. The antipyretic activity of xylopic acid in this model may be attributed to its negative effect on cytokines. This partly may explain the antimalarial effect of xylopic acid.

## 5. Conclusion

Xylopic acid possesses curative and prophylactic properties on *P. berghei*-induced malaria in ICR mice as well as antipyretic properties. It is therefore an ideal antimalarial drug candidate.

## Figures and Tables

**Table 1:** Summary of the effect of XA and A-L on established *Plasmodium berghei* infection in mice.

Parameters	Control (vehicle)	Xylopic acid (mg/kg)			A-L (mg/kg)
		10	30	100	4
Survival days	12 ± 0.4	25 ± 0.3	19 ± 1.3	29 ± 0.8	28 ± 0.2

Values are expressed as mean ± S.E.M. (*n* = 6).

## References

[1] WHO (2010). *World Malaria Report*.

[2] Mishra K, Dash AP, Swain BK, Dey N (2009). Anti-malarial activities of *Andrographis paniculata* and *Hedyotis corymbosa* extracts and their combination with curcumin. *Malaria Journal*.

[3] Randrianarivelojosia M, Rasidimanana VT, Rabarison H (2003). Plants traditionally prescribed to treat tazo (malaria) in the eastern region of Madagascar. *Malaria Journal*.

[4] Basco LK, Mitaku S, Skaltsounis A-L (1994). In vitro activities of furoquinoline and acridone alkaloids against *Plasmodium falciparum*. *Antimicrobial Agents and Chemotherapy*.

[5] Suleiman MM, Mamman M, Aliu YO, Ajanusi JO (2005). Anthelmintic activity of the crude methanol extract of *Xylopia aethiopica* against *Nippostrongylus brasiliensis* in rats. *Veterinarski Arhiv*.

[6] Tatsadjieu LN, Essia Ngang JJ, Ngassoum MB, Etoa F-X (2003). Antibacterial and antifungal activity of *Xylopia aethiopica, Monodora myristica, Zanthoxylum xanthoxyloides* and *Zanthoxylum leprieurii* from Cameroon. *Fitoterapia*.

[7] Woode E, Ameyaw EO, Boakye-Gyasi E, Abotsi WKM (2012). Analgesic effects of an ethanol extract of the fruits of *Xylopia aethiopica* (Dunal) A. Rich, (Annonaceae) and the major constituent, xylopic acid in murine models. *Journal of Pharmacy and BioAllied Sciences*.

[8] Cavalcanti BC, Bezerra DP, Magalhães HIF (2009). Kauren-19-oic acid induces DNA damage followed by apoptosis in human leukemia cells. *Journal of Applied Toxicology*.

[9] National Institute of Health Guidelines for the Care and Use of Laboratory Animals (1996). *Guide for care and use of laboratory animals*.

[10] Ishih A, Suzuki T, Hasegawa T, Kachi S, Wang H, Terada M (2004). *In vivo* evaluation of combination effects of chloroquine with cepharanthin or minocycline hydrochloride against blood-induced choloquine-resistant *Plasmodium berghei* NK65 infections. *Tropical Medicine and Health*.

[11] Al-Adhroey AH, Nor ZM, Al-Mekhlafi HM, Mahmud R (2010). Ethnobotanical study on some Malaysian anti-malarial plants: a community based survey. *Journal of Ethnopharmacology*.

[12] Peters W (1965). Drug resistance in *Plasmodium berghei* Vincke and Lips, 1948. III. Multiple drug resistance. *Experimental Parasitology*.

[13] Santos FA, Rao VSN (1998). A study of the anti-pyretic effect of quinine, an alkaloid effective against cerebral malaria, on fever induced by bacterial endotoxin and yeast in rats. *Journal of Pharmacy and Pharmacology*.

[14] Depinay N, Franetich JF, Grüner AC (2011). Inhibitory effect of TNF-*α* on malaria pre-erythrocytic stage development: influence of host hepatocyte/parasite combinations. *PLoS One*.

[15] Hansen DS (2012). Inflammatory responses associated with the induction of cerebral malaria: lessons from experimental murine models. *PLoS Pathogens*.

[16] Golenser J, Waknine JH, Krugliak M, Hunt NH, Grau GE (2006). Current perspectives on the mechanism of action of artemisinins. *International Journal for Parasitology*.

[17] German PI, Aweeka FT (2008). Clinical pharmacology of artemisinin-based combination therapies. *Clinical Pharmacokinetics*.

[18] Kokwaro G, Mwai L, Nzila A (2007). Artemether/lumefantrine in the treatment of uncomplicated *falciparum* malaria. *Expert Opinion on Pharmacotherapy*.

[19] Haque A, Best SE, Amante FH (2011). High parasite burdens cause liver damage in mice following Plasmodium berghei ANKA infection independently of CD8^+^ T cell-mediated immune pathology. *Infection and Immunity*.

[20] Petersen I, Eastman R, Lanzer M (2011). Drug-resistant malaria: molecular mechanisms and implications for public health. *FEBS Letters*.

[21] Hill DR, Baird JK, Parise ME, Lewis LS, Ryan ET, Magill AJ (2006). Primaquine: report from CDC expert meeting on malaria chemoprophylaxis I. *The American Journal of Tropical Medicine and Hygiene*.

[22] Dow GS, Magill AJ, Ohrt C (2008). Clinical development of new prophylactic antimalarial drugs after the 5th Amendment to the Declaration of Helsinki. *Therapeutics and Clinical Risk Management*.

[23] Ryley JF, Peters W (1970). The antimalarial activity of some quinolone esters. *Annals of Tropical Medicine and Parasitology*.

